# Sequence, structure, and function of the Dps DNA-binding protein from *Deinococcus wulumuqiensis* R12

**DOI:** 10.1186/s12934-022-01857-7

**Published:** 2022-07-02

**Authors:** Yao Chen, Zhihan Yang, Xue Zhou, Mengmeng Jin, Zijie Dai, Dengming Ming, Zhidong Zhang, Liying Zhu, Ling Jiang

**Affiliations:** 1 College of Food Science and Light Industry, State Key Laboratory of Materials-Oriented Chemical Engineering, Nanjing Tech University, Nanjing, 211816 China; 2 College of Biotechnology and Pharmaceutical Engineering, Nanjing Tech University, Nanjing, 211816 China; 3 School of Chemistry and Molecular Engineering, Nanjing Tech University, Nanjing, 211816 China; 4 Institute of Applied Microbiology, Xinjiang Academy of Agricultural Sciences/Xinjiang Key Laboratory of Special Environmental Microbiology, Ürümqi, 830091 Xinjiang China

**Keywords:** *Deinococcus wulumuqiensis* R12, DNA-binding protein, Oxidative stress, Proteomics

## Abstract

*Deinococcus wulumuqiensis* R12, which was isolated from arid irradiated soil in Xinjiang province of China, belongs to a genus that is well-known for its extreme resistance to ionizing radiation and oxidative stress. The DNA-binding protein Dps has been studied for its great contribution to oxidative resistance. To explore the role of Dps in *D. wulumuqiensis* R12, the Dps sequence and homology-modeled structure were analyzed. In addition, the *dps* gene was knocked out and proteomics was used to verify the functions of Dps in *D. wulumuqiensis* R12. Docking data and DNA binding experiments in vitro showed that the R12 Dps protein has a better DNA binding ability than the Dps1 protein from *D. radiodurans* R1. When the *dps* gene was deleted in *D. wulumuqiensis* R12, its resistance to H_2_O_2_ and UV rays was greatly reduced, and the cell envelope was destroyed by H_2_O_2_ treatment. Additionally, the qRT-PCR and proteomics data suggested that when the *dps* gene was deleted, the catalase gene was significantly down-regulated. The proteomics data indicated that the metabolism, transport and oxidation–reduction processes of *D. wulumuqiensis* R12 were down-regulated after the deletion of the *dps* gene. Overall, the data conformed that Dps protein plays an important role in *D. wulumuqiensis* R12.

**Supplementary Information:**

The online version contains supplementary material available at 10.1186/s12934-022-01857-7.

## Introduction

In 1956, a bacterium that had survived exposure to an extremely high dose of ionizing radiation (IR) was accidentally discovered as a contaminant in a can of supposedly sterilized meat [1]. Now well-known as *Deinococcus radiodurans*, it is one of the most radiation-resistant organisms known to science [2, 3]. It is not only tolerant to gamma radiation, but also to other DNA damage and oxidative stress-generating conditions such as UV, desiccation, or high temperature [4–6]. The radiation tolerance of *D. radiodurans* can reach 15,000 Gy [7], which is 100-fold that of typical microorganisms, 250-fold that of *Escherichia coli,* and 3000-fold that of humans [8, 9]. Therefore, *D. radiodurans* is an ideal model strain for studying the oxidative stress response and radiation resistance [10, 11].

Several studies have investigated the remarkable oxidative resistance mechanisms of this bacterium, which can be divided into three categories, indicating DNA self-repair [4], efficient cell evolution mechanism [10], and effective scavenging of reactive oxygen species (ROS) [11–13]. Among them, the DNA repair system of *D. radiodurans* has been identified as a major determinant of its IR resistance. In addition, studies have shown that protein damage is just as important as the DNA damage following exposure to IR. Daly et al. also put forward the viewpoint that proteins are important macromolecule that can be affected by IR [14].

DNA-binding protein (Dps), a conserved protein found in most bacterial species, has been devoted a great deal of attention, since it is a vital factor that protects DNA from various oxidative damage in stressed or starved cells [3, 15, 16]. Recent studies have painted a clearer picture of the two mechanisms through which Dps exerts its protective effects in cells. Firstly, Dps can effectively bind DNA, thereby physically shielding it from attack by oxygen free radicals [17]. In addition, Dps has ferroxidase activity, which is a key feature that prevents the formation of highly toxic ROS from the reaction of iron (II) with hydrogen peroxide or dioxygen [18]. Dps can also be oxidized to protect DNA from a distance by DNA charge transfer (CT), which may be another effective DNA protection mechanism [19]. In agreement with these roles of Dps protein, the *dps* gene is critical for cell survival under stress conditions [20]. When the *dps* gene was knocked out in *Salmonella enterica*, the mutant was more sensitive to antibiotics than the parental strain [21]. Similarly, the *Δdps* mutant of *Riemerella anatipestifer* was more sensitive to H_2_O_2_ under iron-rich conditions [22]. Based on these, we hypothesized that Dps protein or *dps* gene may play a crucial role in the genus *Deinococcus,* and might exhibit unique effects inside the cells.

Here, we studied the functions of Dps from *Deinococcus wulumuqiensis* R12, we used *Deinococcus wulumuqiensis* R12 strain as the experiment material to research, which was previously isolated by our team from a radiation-contaminated area of Xinjiang Uighur Autonomous Region of northwest China. Strain *D. wulumuqiensis* R12 is a Gram-positive, reddish orange, non-spore-forming coccus, which is characterized by extreme gamma radiation resistance of more than 10 kGy and UV resistance more than 700 J/m^2^ [23]. The *D. wulumuqiensis* R12 exhibited higher tolerance to gamma radiation and UV light the prototypical *D. radiodurans* R1, and the genome of *D. wulumuqiensis* R12 has been sequenced in our previous work [23]. The genome revealed a single *dps* gene of 645 bp. In this study, we compared the sequence and structure of Dps from *D. radiodurans* R1 and *D. wulumuqiensis* R12 to clarify the unique functions of Dps from *D. wulumuqiensis* R12. Further, we constructed a *Δdps* mutant of *D. wulumuqiensis* R12 through homologous recombination to explore the *dps*. We combined bioinformatics, protein in vitro assays, genetic engineering and proteomics to explore the functions of *D. wulumuqiensis* R12 Dps in vivo and in vitro. This will provide guidance for the study of proteins without crystal structure using powerful bioinformatic tools and various experimental methods.

## Materials and methods

The *dps* gene was knocked out in *D. wulumuqiensis* R12 which was preserved in our laboratory, using the pK18mobSacB shuttle plasmid from Miaoling Biotechnology Co., Ltd (Wuhan, China). *E. coli* DH5α (Vazyme, Nanjing, China) was utilized for gene cloning. T4 DNA ligase, Phanta DNA polymerase, *Eco*RI and *Bam*HI were purchased from Vazyme (Nanjing, China). Triton X-100, protease inhibitor, TEAB (tetraethylammonium bromide), trypsin, DTT (dithiothreitol), and IAA (iodoacetamide) were from Sangon (Shanghai, China). Yeast extract and tryptone were purchased from Oxoid (UK). Formic acid, acetonitrile, actone, and other chemicals were from Sigma-Aldrich (Shanghai, China). Tryptone glucose yeast (TGY) medium (5 g/L tryptone, 3 g/L yeast extract, and 1 g/L glucose, pH 7.0) was used for *D. wulumuqiensis* R12 culture. Nutrient agar (NA) medium (10 g/L tryptone, 3 g/L beef extract, 5 g/L NaCl, and 15 g/L agar) was utilized for preparation and transformation of competent cells. Luria–Bertani (LB) medium (5 g/L yeast extract, 10 g/L tryptone, and 10 g/L NaCl, pH 7.0) was used for *E. coli* DH5ɑ culture. When needed, 1.5% agar was added to obtain a solid medium.

### Model building of *Deinococcus radiodurans* R1 Dps1 (R1 Dps1) or *Deinococcus wulumuqiensis* R12 Dps (R12 Dps) protein and protein/DNA docking

The R12 Dps structure was modeled using RoseTTAFold, and the N-terminal of R1 Dps structure (PDB code: 2C2F) was reconstructed using the same method. The structures were presented and analyzed using PyMol.

The DNA model was downloaded from PDB. The protein/DNA docking was carried out using AutoDock 4.2.6, and the complex with the lowest energy was selected for further analyses.

### Plasmid construction

The primers used in this study are listed in Additional file 4: Table S1 and the *D. wulumuqiensis* R12 genome was used as the template. The PCR temperature program was as follows: 1 cycle of 300 s at 98 °C, 35 cycles of 60 s at 98 °C, 60 s at 62.5 °C, 60 s at 72 °C, 1 cycle of 600 s at 72 °C. The temperature program for gene splicing by overlap extension PCR (SOE PCR) was follows: 1 cycle of 300 s at 94 °C, 35 cycles of 20 s at 94 °C, 60 s at 61 °C, 30 s at 72 °C, 1 cycle of 600 s at 72 °C. The amplified fragments and pK18mobSacB plasmid were digested with *Eco*RI and *Bam*HI for 4 h at 37 °C, purified and ligated using T4 ligase at 4 °C for 12 h.

The R12 *dps* gene was amplified from the *D. wulumuqiensis* R12 genome, and the primers listed in Additional file 4: Table S1. The PCR temperature program was as follows: 1 cycle of 300 s at 95 °C, 35 cycles of 15 s at 95 °C, 15 s at 65 °C, 60 s at 72 °C, 1 cycle of 600 s at 72 °C. The R12 *dps* fragments were extracted using the “FastPure Gel DNA Extraction Mini Kit (Vazyme)”. The purified fragments were double-digested with *Nde*I/*Xho*I, and ligated T4 ligase to obtain the recombinant plasmid. The Dps1 gene ***D. radiodurans R1*** was colned using the same method to construct the recombinant plasmid.

### Expression and purification of the R12 Dps and R1 Dps1 protein

R12 Dps and R1 Dps1 were expressed in *E. coli* BL21 (DE3) using the vector was pET-22b(+). The recombinant strains were cultured in 50 mL LB medium at 37 °C and 200 rpm till the OD_600_ reached to 0.6–0.8. Then, IPTG was added to a final concentration of 0.5 mM to induce the protein expression, which was continued at 200 rpm for overnight at 20 °C.

The cells from 50 mL culture were harvested by 8000×*g* centrifugation for 5 min, and the discarded the supernatants. Then, the cells were resuspended with 3 mL PBS buffer. The cells were disrupted by sonication at 300 W for 15 min, and centrifuged at 10,000×*g* for 20 min at 4 °C. The supernatants were obtained and loaded onto 1 mL Ni–NTA resin that was pre-equilibrated with 5 mL buffer A (pH 8.0) that contains 20 mM imidazole and 300 mM NaCl. Then, 5 mL buffer A was used to remove non-specifically bound proteins. Finally, 3 mL buffer B containing 300 mM imidazole and 300 mM NaCl (pH 8.0) was used to elute the target proteins. The supernatant and purified proteins were analyzed by SDS polyacrylamide gel electrophoresis (SDS-PAGE), and the protein concentration was measured using a nano-spectrophotometer (Colibri, Germany).

### The DNA protection effect of R12 Dps and R1 Dps1 protein

The pET-22b-R12-Dps and pET-22b-R1-Dps1 recombinant plasmids were constructed using the primers listed in Additional file 4: Table S1. Samples comprising 40 μM purified R12 Dps and R1 Dps1 protein in double distilled water and crosslinker fluid (pH 8.0 20 mM phosphate buffer with 80 mM NaCl and 0.1% glutaraldehyde) were incubated at room temperature for 30 min. Then, 10 μL pET-22b plasmids (60 ng/μL) and 10 μL 40 μM R12 Dps or R1 Dps1 protein were mixed and incubated at room temperature for 30–60 min. Samples containing 30 ng/μL plasmids DNA were using as control. Finally, agarose gel electrophoresis was utilized to verify the protective effect of Dps proteins.

### Validation of catalase expression by qRT-PCR

The WT R12 strain and *Δdps* R12 mutant were grown in TGY medium at 31 °C for 3 days. The 4 mL cells were harvested by centrifugation at 12,000×*g* for 3 min. The total RNA was isolated using the bacteria total RNA isolation kit (Sangon, Shanghai). The cDNA was obtained using one-step gDNA removal and cDNA synthesis SuperMix (TransGen Biotech, Beijing). The real-time PCR was carried out on a Roche LightCycler96 real-time fluorescence quantitative PCR instrument. The 16S rRNA gene was used as the internal reference. The primers used for qRT-PCR are listed in Additional file 4: Table S1.

### Growth curve analysis of WT R12 strain and the *Δdps* R12 mutant

The WT R12 strain and *Δdps* R12 mutant at the logarithmic growth stage were transferred into 50 mL of TGY liquid medium, at an inoculation amount of 2%, after which the OD_600_ value was measured every 2 h. Each sample was measured in triplicate and the mean value was recorded.

### Survival rate of WT R12 strain and *Δdps* R12 mutant under oxidative stress

The WT R12 strain or *Δdps* R12 mutant was cultured for 48 h in TGY liquid medium at 31 °C. The resulting seed culture was used to inoculate, fresh TGY medium to an initial OD_600_ of 0.6–0.8. Next, the cells were treated with 80 mM H_2_O_2_ for 0, 10, 20, 30, and 50 min at 31 °C and 800 rpm in a heating block. After the stress treatment, 100 μL (10^9^ CFU) aliquots of serial tenfold dilutions (10^–1^–10^–5^) of cells were plated onto TGY agar and grown at 31 °C for 3 days. The survival rate was calculated based on the number of colonies in the treated samples compared with the untreated sample (control group). The 10^–5^ dilution was plated onto TGY agar to calculate the number of colony-forming units (CFU) in triplicate.

### Survival rate of the WT R12 strain and *Δdps* R12 mutant after exposure to UV irradiation

The *Δdps* R12 mutant was cultured to the stationary stage and diluted in a 10^6^-fold gradient. Then, 200 μL (10^6^ CFU) of the diluted cell suspension were plated onto TGY agar, exposed to 0, 3, 6, 9 and 12 min 700 J/m^2^ UV irradiation, and cultured for 2–3 days at 31 °C. The survival rate was calculated after counting the colonies. Each group included three independent repeats. The WT R12 strain was included under the same conditions as the control group.

### Transmission electron microscopy (TEM)

The WT R12 strain and *Δdps* R12 mutant were grown in TGY liquid medium at 31 °C to an OD_600_ value of 0.6–0.8, at which point they were treated with 80 mM H_2_O_2_ for 30 min at 31 °C. Then, the cells were washed twice with PBS and collected by centrifugation at 12,000×*g* for 2 min. The cells were fixed overnight at 4 °C with 2.5% glutaraldehyde, harvested by centrifugation at 4000×*g* for 5 min, and embedded in 2% agarose. The slices were stained with uranyl acetate for 15 min and observed under a Hitachi H-7650 transmission electron microscope.

### Protein extraction and digestion

Samples comprising 200 μg of cells from a culture with an OD_600_ of 0.8 were shock frozen − 80 °C. The cells were weighed into a pre-cooled mortar, and liquid nitrogen was added. Then, 4 times the volume of lysis buffer (1% Triton X-100 and 1% protease inhibitor) was added to each sample. The cells were disrupted by sonication (240 W) for 15 min. After centrifugation at 12,000×*g* for 10 min, the supernatant was transferred to a fresh centrifuge tube and the protein concentration was determined using a BCA assay kit (Sangon, Shanghai, China). An equal amount of each sample was subjected to enzymatic hydrolysis, and the volume was adjusted with lysis buffer. Then, 1 volume of pre-cooled acetone was added and vortexed, after which fourfold pre-cooled acetone was added. The precipitation took place at − 20 °C for 2 h. After centrifugation at 4500×*g* for 5 min, the supernatant was discarded and the precipitate was washed twice with pre-cooled acetone. After drying and precipitation, TEAB with a final concentration of 200 mM was added, followed by ultrasonic dispersion of the precipitate. Trypsin was added at a ratio of 1:50 (protease:protein, m/m) and enzymatic hydrolysis was conducted overnight. DTT was added to a final concentration of 5 mM and incubated at 56 °C for 30 min. Then, IAA was added to a final concentration of 11 mM and incubated at room temperature in the dark for 15 min. Finally, the peptides were desalted using a C18 SPE column. Each strain was grown in triplicate cultures, and each sample was treated as described above.

### Liquid chromatography tandem mass spectrometry (LC–MS/MS) analysis

The peptide segments were separated by UPLC on a NanoElute instrument (Bruker Daltonics) mobile phase A and then separated using a NanoElute ultra-high performance liquid phase system. Mobile phase A was water with 0.1% formic acid and 2% acetonitrile. Mobile phase B was acetonitrile with 0.1% formic acid and 100%. The mobile phase gradient settings were as follows: 0–70 min, 4–22% B; 70–84 min, 22–30% B; 84–87 min, 30–80% B; 87–90 min, 80% B. The flow rate maintained was 450 nL/min. The peptide segments were separated by UPLC and then ionized by injecting into the capillary ion source. The peptide segments were analyzed using a timsTOF Pro (Bruker Daltonics) mass spectrometry instrument. Mass spectrometry data was acquired by Bruker Compass HyStar (Version 5.1.8.1), and analyzed by MaxQuant 1.6.6.0. The voltage of the ion source was set to 1.75 kV, and the parent ion of the peptide segments and its secondary fragments were detected and analyzed using high-resolution TOF scanning. The scanning range of secondary mass spectrometry was set to 400–1500 m/z. The data acquisition was conducted in parallel cumulative serial fragmentation (PASEF) mode. After one set of first-order mass spectrometry data was acquired, secondary spectrographs with the charge of the parent ion in the range of 0–5 were collected in PASEF mode 10 times. The dynamic elimination time of tandem mass spectrometry scanning was set to 30 s to avoid repeated scanning of the parent ion.

In our results, at least one razor or unique peptide of a protein was to be considered as identified, the minimum score of peptides was set as 40, and the false discovery rate (FDR) was set to 1% to ensure the identities are authentic. FDR is a measure of the incorrect peptide spectral matches (PSMs) among all accepted PSMs [24–26]. Proposed by Benjamini and Hochberg [27] as an alternate to the Bonferroni correction, it is defined as the rate of false positives among accepted hits. FDR is a less stringent metric for global confidence assessment. In the context of proteomics, it is a global estimate of the false positives present among the results obtained by a database search algorithm [28].

### Differential protein screening

Protein difference analysis first picks out the samples to be compared, calculates the quantitative mean proteins of the repeated samples, and finally calculates the difference multiple of the comparison group was calculated. The calculation formula is shown in ɑ. In order to judge the significance of the differences, Student’s *t*-test was applied to the relative quantitative value of each protein in the two comparison samples, and the corresponding *P* value was calculated, which was taken as the significance index. The default *P* value was ≤ 0.05. To make the test data conform to the normal distribution, the relative quantitative values of proteins need to undergo log2 logarithmic conversion before testing. The calculation formula is shown in β. Through the above difference analysis, when *P* ≤ 0.05, the change of differential expression level over 1.5 was regarded as the change threshold of significantly up-regulated, and less than 1/1.5 was regarded as the change threshold of significantly down-regulated proteins. The summary data of all differentially expressed proteins in this project are shown in “MS_identified_information” (Additional file 3).

- ɑ: FC {A/B,k} = Mean(R {ik},i\in A)/Mean(R {ik}, i\in B)
- β: [P {ik} = T.test(Log2(P {ik},i\in A),Log2(P {ik}, i\in B))\]

A and B are the samples, R represents the relative quantity of protein, i represents the sample, and k represents the protein.

### GO and KEGG enrichment analyses

The GO and KEGG enrichment analyses were performed using Blast2go (version 5.2, Biobam, Valencia, https://www.blast2go.com/) and DAVID (version 6.8, https://david.ncifcrf.gov), respectively. GO terms and KEGG pathways with corrected *P*-values of less than 0.05 were considered to be significantly enriched among the identified differentially expressed proteins (Additional files 1, 2).

### Prediction of protein–protein interactions

The number of sequences of the differentially expressed-proteins screened according to the fold change over 1.5 in different comparison groups was compared with the protein network of the STRING database (v.11.0), and the interaction of the partners of the differentially expressed proteins were extracted according to a confidence score > 0.7 (high confidence). “Cytoscape 3.8.2” software was used to visualize the interaction network of differentially expressed proteins [29]. The “network.links.txt” was imported into Cytoscape. Then, the “network.nodes.txt” was loaded into “network.links.txt”. The layout was set with “Preduse Force Directed Layout”. The node of fill color coloum set “Log2Ratio”, the mapping type was “Continuous Mapping”.

## Results and discussion

### Sequence and structure analyses of *D. wulumuqiensis* R12 Dps

**Fig. 1:**
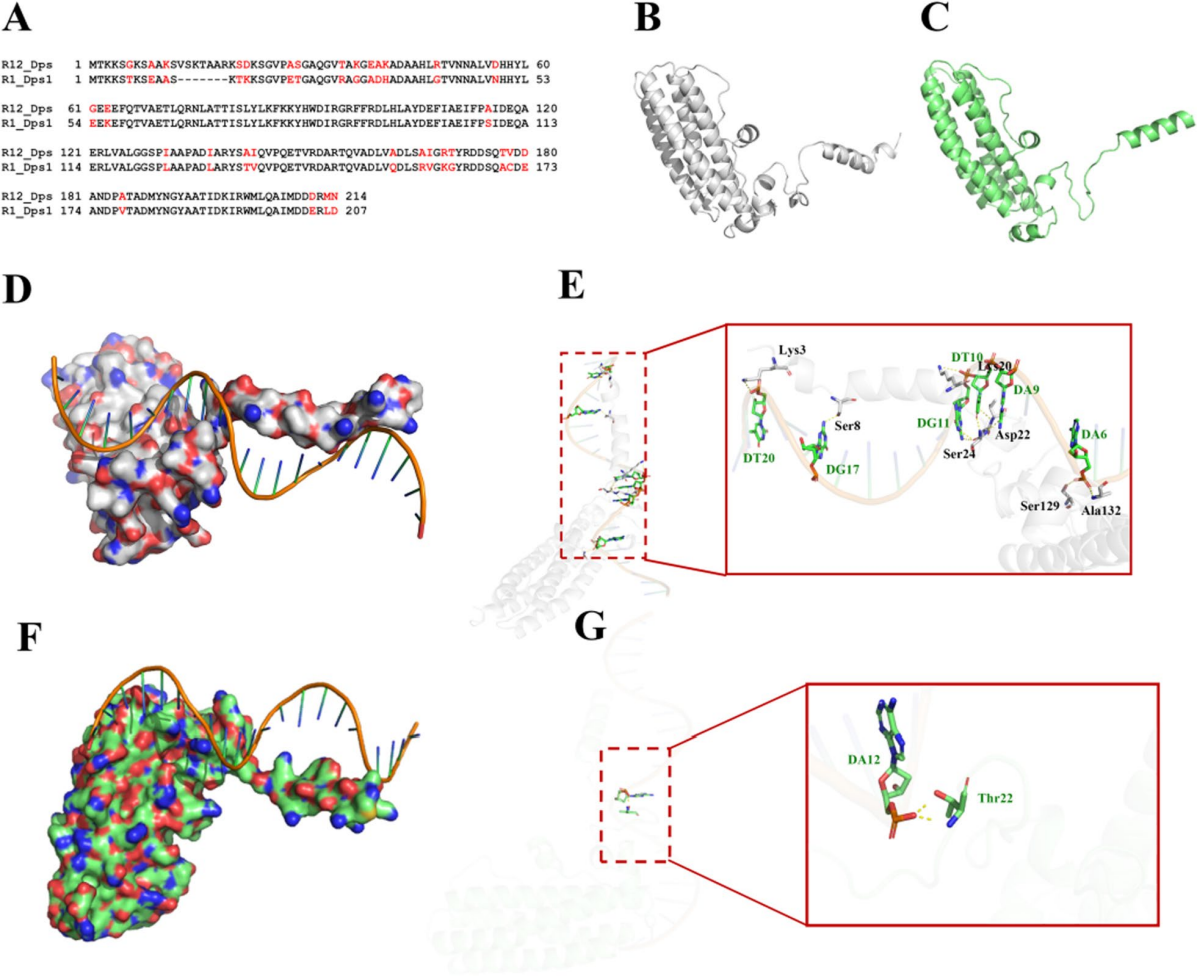
Sequence alignment and structure comparison of R12 Dps and R1 Dps1. **A** The sequence alignment between R12 Dps and R1 Dps1. **B** R12 Dps model constructed using RoseTTAFold. **C** The R1 Dps1 model which was repaired by RoseTTAFold which the original structure was 2C2F (PDB code). **D** The docking result between R12 Dps and DNA. **E** Hydrogen bonds between R12 Dps protein and DNA. **F** The docking result of R1 Dps1 and DNA. **G** Hydrogen bonds between R1 Dps1 and DNA

To explore the function of *D. wulumuqiensis* R12 Dps, the sequence (Fig. 1A) and structure were analyzed. Through sequence alignment, Dps1 from *Deinococcus radiodurans* R1 (PDB code: 2C2F, missing the N-terminus) was identified as having the highest identity (81.31%), and the alignment between R12 Dps and R1 Dps1 sequences was carried out. The alignment result showed that the N-terminal sequences of R1 Dps1 and R12 Dps were not conserved, and might play an important role in functional differences. Recent research articles have reported that the N-terminal lysine residues of Dps proteins from *E. coli* have are involved in DNA binding [30].

In order to verify the function of the N-terminus of R12 Dps protein. The R12 Dps model was built using RoseTTAFold (Fig. 1B) [31]. The N-terminus of R1 Dps1 crystal structure was too flexible and could not be analyzed, therefore. Therefore, RoseTTAFold was used to reconstruct the missing part (Fig. 1C). The structures of the main body of R1 Dps and R12 Dps were similar, with an α-helical secondary structure. The N-termini exhibited obvious difference that might determine the functional differences. Furthermore, protein/DNA docking was carried out using AutoDock 4.2.6 (Fig. 1D–G) [32]. The docking results showed that the DNA binding ability of R12 Dps was better than that of R1 Dps1. The Lys3, Ser8, Ser20, Lys20, Asp22, Ser129 and Ala132 residues interacted with the DNA via hydrogen bonds in the R12 Dps/DNA docking result, while in the R1 Dps1/DNA docking result, only Thr22 directly interacted with the DNA. Previous studies clarified that the N-terminus domain plays a crucial role in DNA-binding [15, 33]. The docking data confirmed that R12 Dps has a better DNA-binding ability than R1 Dps1.

### Purification of Dps proteins, DNA binding and protection assays

**Fig. 2:**
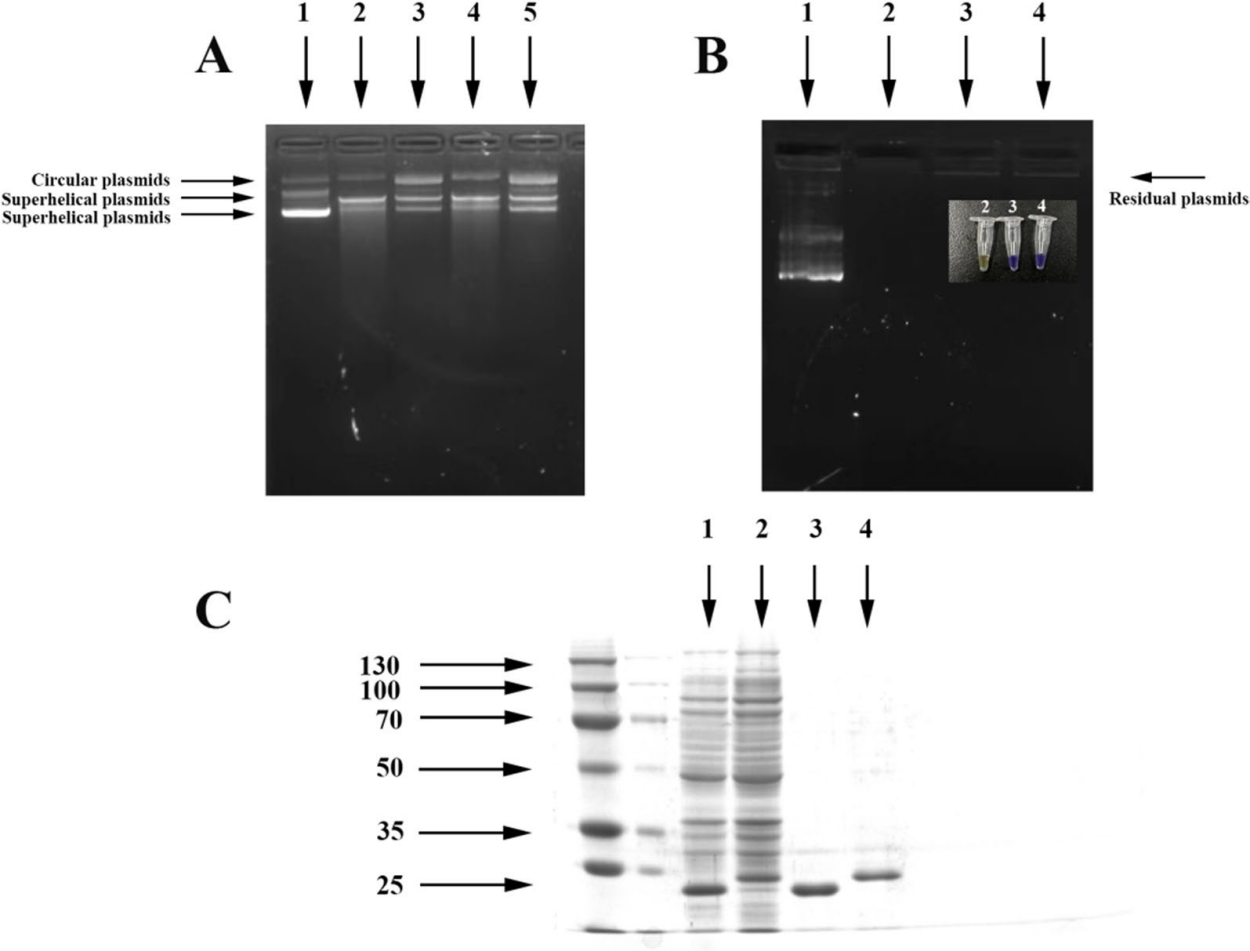
The purification of R12 Dps and R1 Dps1 proteins. **A** DNA binding assays with R12 Dps and R1 Dps1 proteins. Lane 1: pET-22b(+) plasmid; Lane 2: The plasmid combined with R1 Dps1 protein (in deionized water); Lane 3: The plasmid combined with R12 Dps protein (in deionized water); Lane 4: The plasmid combined with R1 Dps1 proteins (crosslinked with 0.1% glutaraldehyde); Lane 5: The plasmid combined with R12 Dps protein (crosslinked with 0.1% glutaraldehyde). **B** DNA protection experiments with R12 Dps and R1 Dps1 proteins. Lane 1: pET-22b(+) plasmid; Lane 2: The plasmid following exposure to 80 mM H_2_O_2_; Lane 3: The resulting plasmid following exposure 80 mM H_2_O_2_ with the protection of the R1 Dps1 protein; Lane 4: The plasmid following exposure 80 mM H_2_O_2_ with the protection of the R12 Dps protein; **C** The purification of R1 Dps1 and R12 Dps proteins. Lane 1: The crude extract of pET-22b(+)-R1Dps1 recombinant *E. coli.* Lane 2: The crude extract of pET-22b(+)-R12Dps recombinant *E. coli.* Lane 3: The purified R1 Dps1 protein; Lane 4: The purified R12 Dps protein

The docking results showed that the DNA binding ability of R12 Dps was better than that of R1 Dps1. The DNA binding assay was used to confirm this computational result. The *dps* gene was amplified from the genome of *D. wulumuqiensis* R12, and the recombinant plasmid pET-22b(+)-R12Dps was constructed for expression in *E.coli* BL21 (DE3). The same method was utilized to obtain the pET-22b(+)-R1Dps1 plasmid which the genomic DNA of *D. radiodurans* R1 as the template. The SDS-PAGE result (Fig. 2C) showed that R1 Dps1 and R12 Dps proteins can be successfully expressed and purified. The molecular weight of R1 Dps1 was ~ 24 kDa, and that of R12 Dps was ~ 25 kDa, which was consistent with the respective theoretical values.

As shown in Fig. 2A, the plasmids DNA of pET-22b(+) appeared superhelical in vitro. The superhelicity of the plasmid was reduced with the addition of R1 Dps1 or R12 Dps proteins. The lower superhelicity of plasmid DNA with added R12 Dps indicated that the DNA binding ability of the R12 Dps was indeed better than that of R1 Dps1, in agreement with the docking data.

The Dps protein can bind Fe^2+^ ions to prevent Fenton reaction [4]. Therefore, 80 mM H_2_O_2_ and 200 μM FeSO_4_ were added to the plasmid DNA. As shown in Fig. 2B, the plasmid DNA was destroyed without the addition of Dps proteins, while the plasmids combined with the R1 Dps1 or R12 Dps proteins were protected to a certain extent, indicating that the Dps protein can bind Fe^2+^ and prevent the Fenton reaction. Additionally, bromophenol blue was oxidized into a yellow product via the Fenton reaction, while the solution with added Dps proteins maintained the blue color, demonstrating that the Dps protein can bind Fe^2+^. These data showed that both R1 Dps1 and R12 Dps have significant DNA binding and protection effects. However, the DNA binding ability of R12 Dps was significantly better than that of R1 Dps1, which might due to the difference of N-terminal.

### Knockout of the *dps* gene in *D. wulumuqiensis* R12

**Fig. 3:**
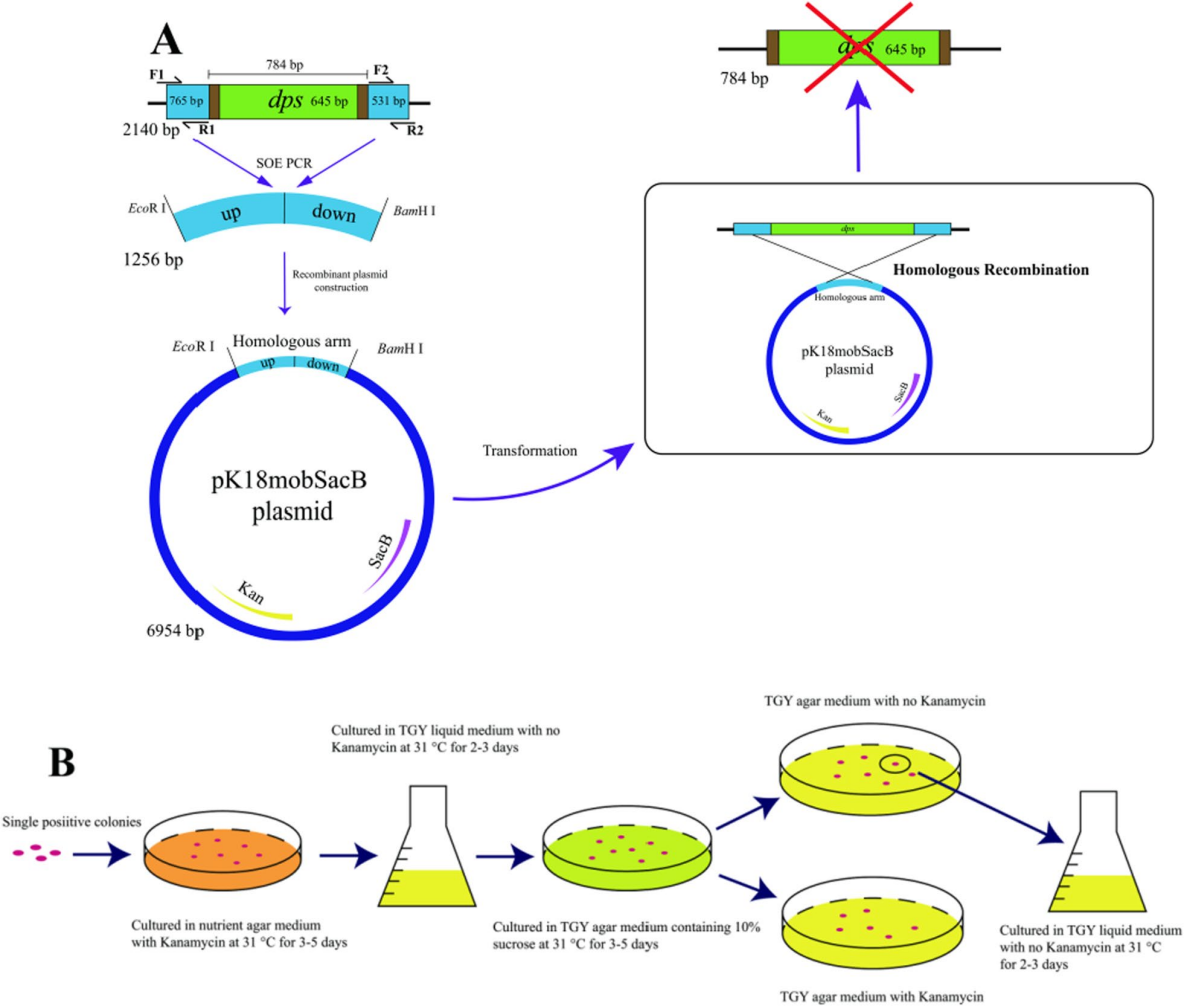
Construction of the *Δdps* R12 mutant. **A** Homologous recombination to knock out the *dps* gene. **B** The screening of *Δdps* R12 mutant colonies

In order to explore the specific functions of Dps in *D. wulumuqiensis* R12, the *dps* gene was knocked out through homologous recombination. In this study, the suicide plasmid pK18mobSacB was utilized as a bifunctional screening vector with kanamycin resistance gene as a positive selection marker, and the *sac*B gene which encodes a secretory levansucrase as a negative selection marker. The recombinant plasmid was used to knock out the *dps* gene through homologous recombination in *D. wulumuqiensis* R12 as shown in Fig. 3A.

The initial screening was conducted on kanamycin plates to obtain the strains containing the recombinant plasmid (Fig. 3B). Then, the secondary screening was carried out, and the single colonies were selected from the sucrose NA plate and streaked onto TGY plates with or without kanamycin to obtain the strains which have been cured of the recombinant plasmid. Clones that grew on the NA plate without kanamycin but not on kanamycin were selected. The genomic DNA of the rescreened knockout and WT R12 strains was isolated, and homologous primers were used for PCR amplification. The genomic DNA of wild-type R12 and *Δdps* R12 mutant was extracted and the target locus was sequenced. The sequencing result with the primers were F1 and R2 showed that a 784 bp segment of the *dps* gene was knocked out in the *Δdps* R12 mutant strain (Additional file 4: Fig. S1). Here, the target we designed to replace the *dps* gene contains additional 139 bp on both sides of the target sequence (645 bp). Finally, the PCR products obtained using primers F1 and R2 (Additional file 4: Table S1) were verified by sequencing, which demonstrated that the *dps* gene was indeed knocked out from R12 genome, resulting in the *Δdps* R12 mutant.

### Growth of *D. wulumuqiensis* R12 after deletion of the *dps* gene

**Fig. 4:**
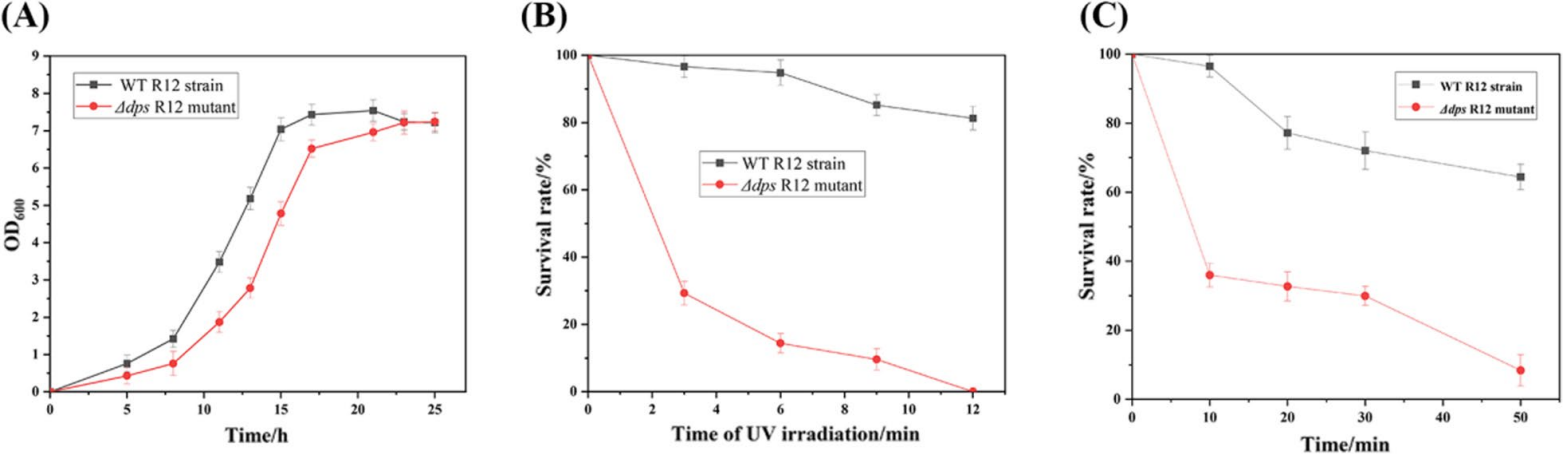
**A** The growth curve of the WT R12 strain and *Δdps* R12 mutant at 31 °C. **B** The survival rate of WT R12 strain and *Δdps* R12 mutant after 0, 3, 6, 9, and 12 min under 700 J/m^2^ UV irradiation. **C** The survival rate of WT R12 strain and *Δdps* R12 mutant after H_2_O_2_ treatment

Dps has been shown to play an important role during exponential phase and stationary phase growth in *E. coli* [3]. In this study, we investigated the effects of the *dps* gene deletion on the growth of *D. wulumuqiensis* R12. As shown in Fig. 4A, the WT R12 strain and *Δdps* R12 mutant both entered the logarithmic phase at 8 h. However, the OD_600_ value of the WT R12 strain was about twofold higher than of the *Δdps* R12 mutant. At 15 h, the WT R12 strain entered the stationary phase with the OD_600_ value reaching ~ 7, while the OD_600_ value of the *Δdps* R12 mutant was less than 5 and it only reached the stationary phase at 21 h. These results indicated that the cell growth of *D. wulumuqiensis* R12 significantly affected by the *dps* gene deletion.

### The survival rate of WT R12 strain and *Δdps* R12 mutant under exposure to UV rays

According to the analyses of Dps structure and sequence, Dps can bind DNA to protect the chromosome via its Dps N-terminal domain. UV irradiation was used to confirm that the UV tolerance of *D. wulumuqiensis* R12 was significantly reduced when Dps protein was lost. As shown in Fig. 4B, after 6 min of UV irradiation, the survival rate of WT R12 strain was more than 90%, while that of the *Δdps* R12 mutant was less than 20%. When the irradiation time reached 12 min, no cells of the *Δdps* R12 mutant were able to survive on the plate, whereas the survival rate of the WT R12 strain was about 80%. These results are in agreement with previous findings that Dps protein plays a crucial role in protecting DNA.

### Sensitivity to H_2_O_2_ oxidative treatments of WT R12 strain and the *Δdps* R12 mutant

The in vitro DNA protection experiment described above confirmed that R12 Dps protein can reduce the Fenton reaction to a certain extent. In order to investigate the role of R12 Dps proteins in vivo, the WT R12 strain and *Δdps* R12 mutant were treated with 80 mM H_2_O_2_ for 0, 10, 20, 30, and 50 min. The survival rate of WT R12 strain and *Δdps* R12 mutant was calculated compared to the untreated sample (0 min treatment). As the H_2_O_2_ treatment time was increased (Fig. 4C), the survival rate of WT R12 strain and *Δdps* R12 mutant decreased the survival rate of the *Δdps* R12 mutant was 35.98% after 10 min of H_2_O_2_ treatment, while the WT R12 strain maintained a survival rate of 96.52%. When the H_2_O_2_ treatment time reached 50 min, the *Δdps* R12 mutant was almost fully inactivated, while the WT R12 strain retained 64.44% survival rate, which was eightfold that of the *Δdps* R12 mutant. These results suggested that the oxidation resistance of *D. wulumuqiensis* R12 was weakened when the *dps* gene was deleted.

### TEM analyses of WT R12 strain and the *Δdps* R12 mutant

**Fig. 5:**
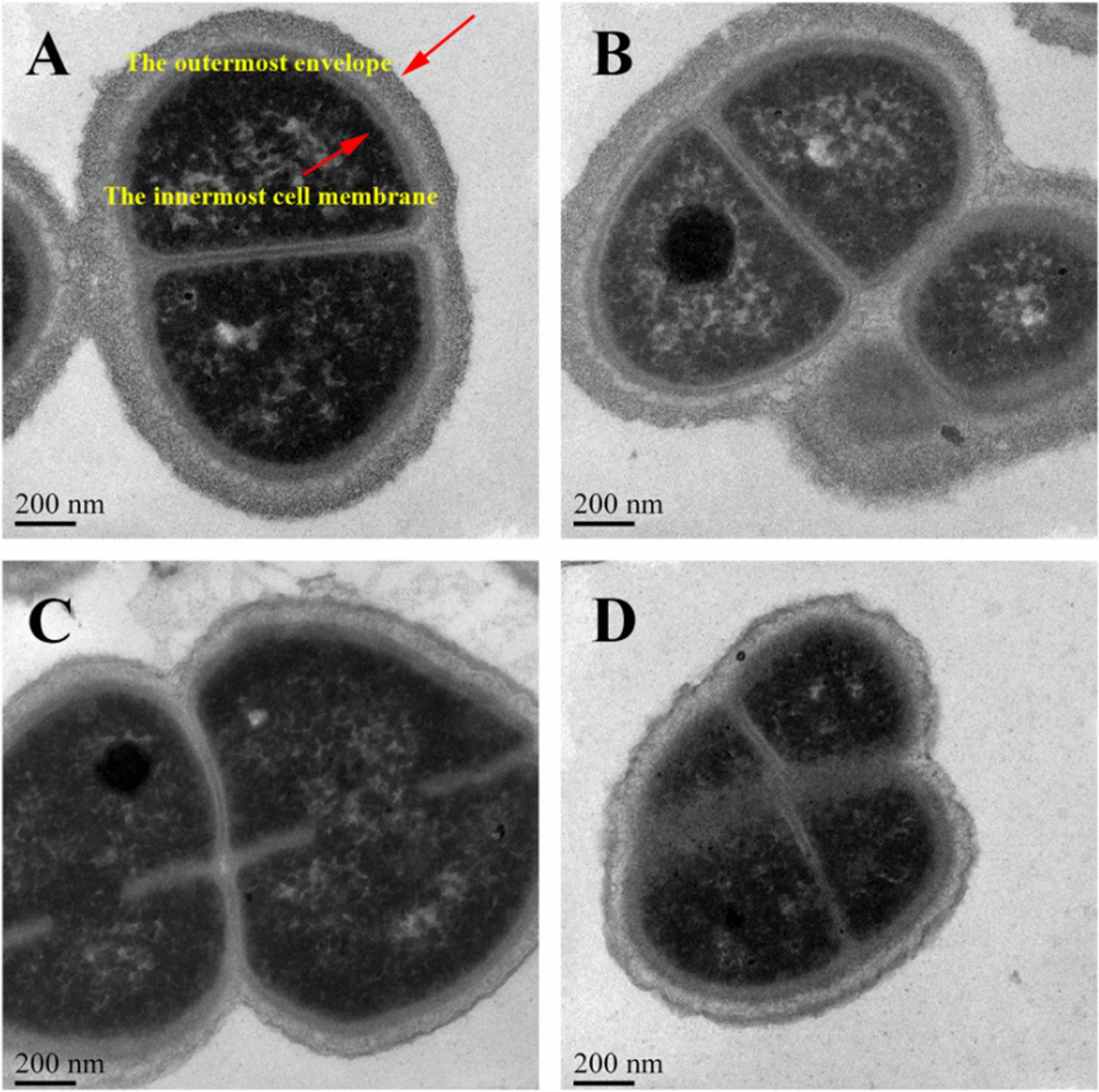
TEM analysis of WT R12 strain and *Δdps* R12 mutant after treatment with 80 mM H_2_O_2_ for 15 min. **A** The WT R12 strain under normal growth conditions. **B** The *Δdps* R12 mutant under normal growth conditions. **C** The WT R12 strain following treatment with H_2_O_2_. **D** The *Δdps* R12 mutant following treatment with H_2_O_2_

The cells of *D. wulumuqiensis* R12 were more sensitive to H_2_O_2_ after the *dps* gene deletion. To explore the specific effects of H_2_O_2_ on the *Δdps* R12 mutant, the WT R12 strain and *Δdps* R12 mutant were both treated with 80 mM H_2_O_2_ for 15 min, after which the cells were fixed for TEM observation. As shown in Fig. 5A and B, the cell envelope thickness of the WT R12 strain and the *Δdps* R12 mutant was approximately 160 nm under normal conditions, and the observable cell envelope was divided into four layers. However, the cell envelope thickness of the WT R12 strain was reduced to about 120 nm after H_2_O_2_ treatment (Fig. 5C), and the cell envelope suffered extensive damage in the *Δdps* R12 mutant (Fig. 5D). The unusually thick cell envelope is the first barrier of *D. wulumuqiensis* R12 against external stresses when faced with a hostile environment. The antioxidant capacity of *D. wulumuqiensis* R12 was significantly reduced when the dps gene was deleted. Previous research showed that the N-terminus of *Dr*Dps2 of *D. radiodurans* can interact with the membrane [34]. We hypothesized that without the protection of Dps protein, the antioxidant capacity of the cell envelope was reduced.

### Difference of proteins expression between the WT R12 strain and the *Δdps* R12 mutant

**Fig. 6:**
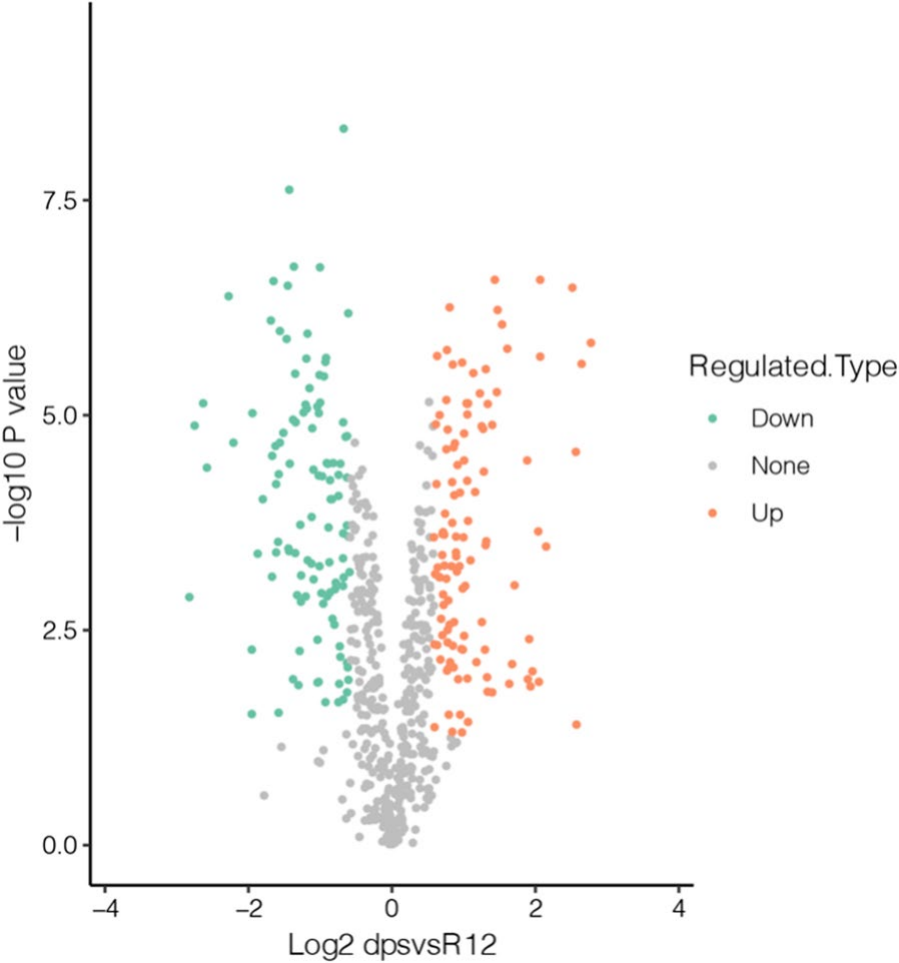
Volcano plot of differentially expressed protein. The relative quantitative value of proteins was subjected to log2 logarithmic conversion, and the *P* value was converted to a log10 value. The proteins detected in the WT R12 strain and the *Δdps* R12 mutant are shown. Each point represents a protein. The orange points indicate up-regulated proteins, and the green points indicate down-regulated proteins in the *Δdps* R12 mutant

For the proteomic analysis, both the WT R12 strain and the *Δdps* R12 mutant were grown in triplicate cultures, and each sample was taken in the logarithmic phase. A total of 1009 proteins were detected by spectrum search analysis. Among these, 116 proteins were up-, and 111 were down-regulated, with fold change > 1.5 and *P* value < 0.05 (Fig. 6). Thus, the numbers of up- and down-regulated proteins following *dps* knockout were similar. In this work, we mainly focused on a series of adverse effects caused by *dps* gene knockout in *D. wulumuqiensis* R12. Therefore, the down-regulated proteins were analyzed further.

### GO analysis of differentially expressed proteins in the WT R12 strain and the *Δdps* R12 mutant

**Fig. 7:**
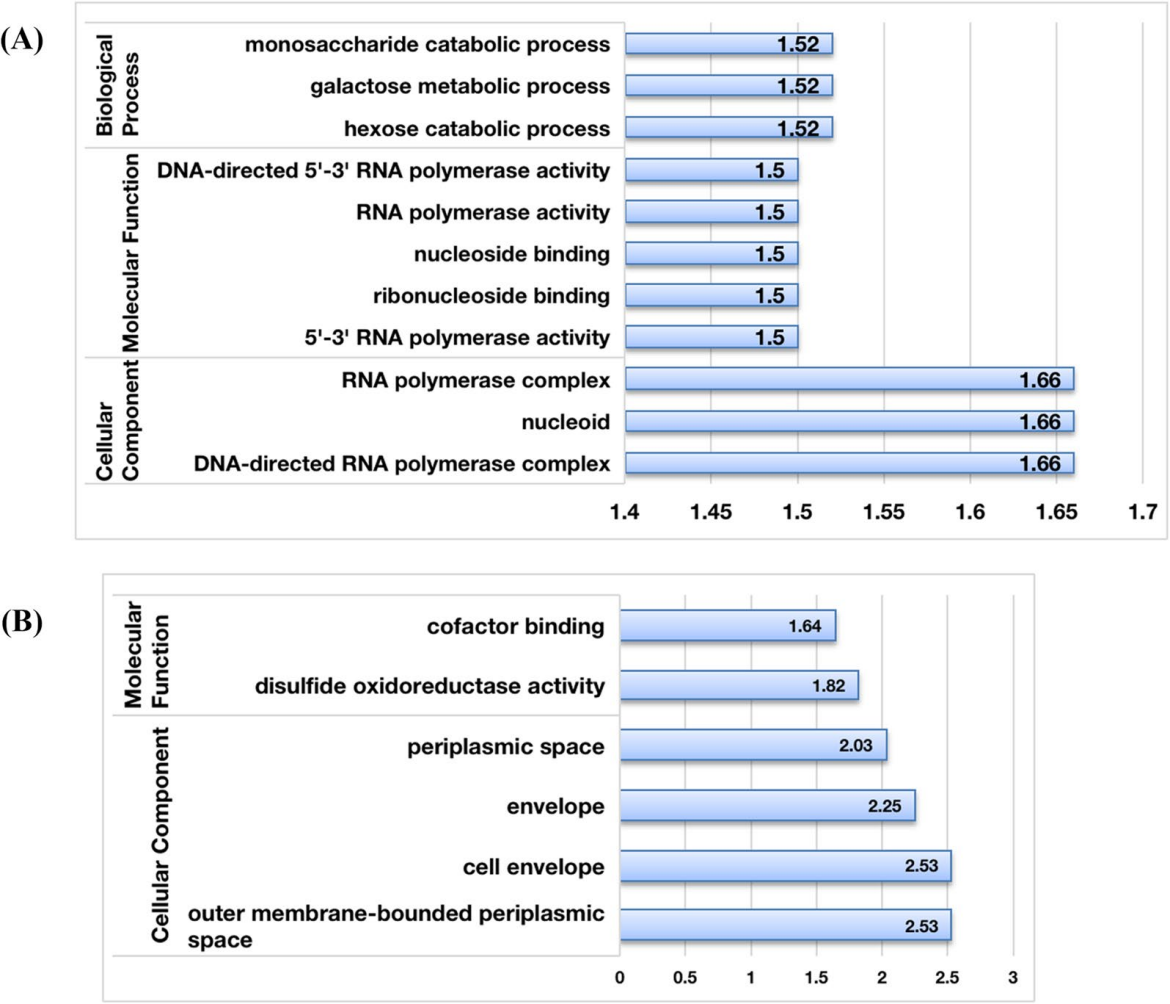
Analysis of differently enriched GO terms between the *Δdps* R12 mutant and WT R12 strain. **A** The up-regulated enrichment GO terms in *Δdps* R12 mutant. **B** The down-regulated enrichment GO terms in the *Δdps* R12 mutant

To further understand the functional characteristics of the differential expressed proteins, GO enrichment analyses of the categories Cellular Component, Molecular Function, and Biological Process were performed. The up-regulated proteins were enriched for the Cellular Component categories DNA-directed RNA polymerase complex, nucleoid, and RNA polymerase. They were also enriched for the molecular functions 5′-3′ RNA polymerase activity, ribonucleoside binding, nucleoside binding, RNA polymerase activity, and DNA-directed 5′-3′ RNA polymerase activity, as well as the Biological Process categories hexose catabolic process, galactose metabolic process and monosaccharide catabolic process (Fig. 7A). The down-regulated proteins were enriched in the Cellular Component categories outer membrane-bound periplasmic space, cell envelope, envelope, and periplasmic space. They were also enriched in the Molecular Function categories disulfide oxidoreductase activity, and cofactor binding (Fig. 7B). Additionally, the down-regulated proteins were enriched in GO terms, metabolic process, cellular process, growth, response to stimulus, interspecies interaction between organisms, localization, multi-organism process, biological regulation in the category Biological Process; cell, intracellular, protein-containing complex in the category Cellular Component; as well as catalytic activity, binding, antioxidant activity, and molecular carrier activity (Additional file 4: Fig. S2A). The up-regulated proteins were enriched in GO terms cellular process, metabolic process, response to stimulus, growth, biological regulation (Biological Process); cell, intracellular, protein-containing complex (Cellular Component); as well as catalytic activity, binding, transporter activity, antioxidant activity, molecular function regulator, and transcription regulator activity (Molecular Function) (Additional file 4: Fig. S2B). These data imply that Dps may influence a large number of proteins with different functions.

The TEM results showed that the cell envelope of the *Δdps* R12 mutant was severely damaged by treatment with 80 mM H_2_O_2_. Cells of *D. wulumuqiensis* R12 can form tetrads, and possess a thick cell envelope (Fig. 5). The cell envelope of *Deinococcus* exhibits an unusual structure and composition [35], and some *Deinococcus* species have six layers in the cell envelope. The entire *D. radiodurans* cell is enveloped by a dense carbohydrate shell, a thick cellular structure that might contribute to its extreme stress resistance [35, 36]. *D. wulumuqiensis* R12 shares similar cell-envelope characteristics, and its envelope is even thicker than that of in *D. radiodurans* [6]. In the *Δdps* R12 mutant, proteins in the GO category cell envelope were significantly down-regulated, indicating the subcategories ABC transporter substrate-binding protein, phosphate-binding protein, and thiamine ABC transporter substrate-binding protein, which might explain why the envelope was more sensitive to H_2_O_2_.

To investigate the response to oxidative stress in *D. wulumuqiensis* R12, several crucial proteins were analyzed. In the Biological Process and Molecular Function categories, under GO term “response to oxidative stress”, enzymes such as catalase (DVJ83_01425), and dihydrolipoyl dehydrogenase (*lpd*A) were significantly down-regulated. Catalase is a metalloenzyme that detoxifies H_2_O_2_ into water and O_2_, thereby protecting organisms from oxidative damage caused by H_2_O_2_ [37]. Also, we found two catalase genes (catalase1 and catalase2) in the genome of *D. wulumuqiensis* R12, and catalase2 was significantly down-regulated (Additional file 4: Fig. S3). These data indicate catalase genes were also affected with the deletion of *dps*, further reducing the H_2_O_2_ tolerance of the *Δdps* R12 mutant.

The growth of the *Δdps* R12 mutant was significantly slower than that of the WT strains. ABC substrate-binding transporter were significantly down-regulated. ABC transporters (ATP-binding cassette transporter) are one of the largest and oldest protein families, which plays a crucial role in the physiology of all organisms [38, 39]. It uses the energy of ATP hydrolysis to transport a wide range of biomolecules across the membrane [39]. The substrate-binding proteins bind the substrate with high affinity and deliver it to the transporter [40]. ABC importers are major determinants of the acquisition of essential nutrients in bacteria [41, 42]. The slow growth of *D. wulumuqiensis* R12 after *dps* gene knock out may be explained by the down-regulation of ABC substrate-binding transporter.

### KEGG analysis of differently expressed proteins in the WT R12 strain and the *Δdps* R12 mutant

**Fig. 8:**
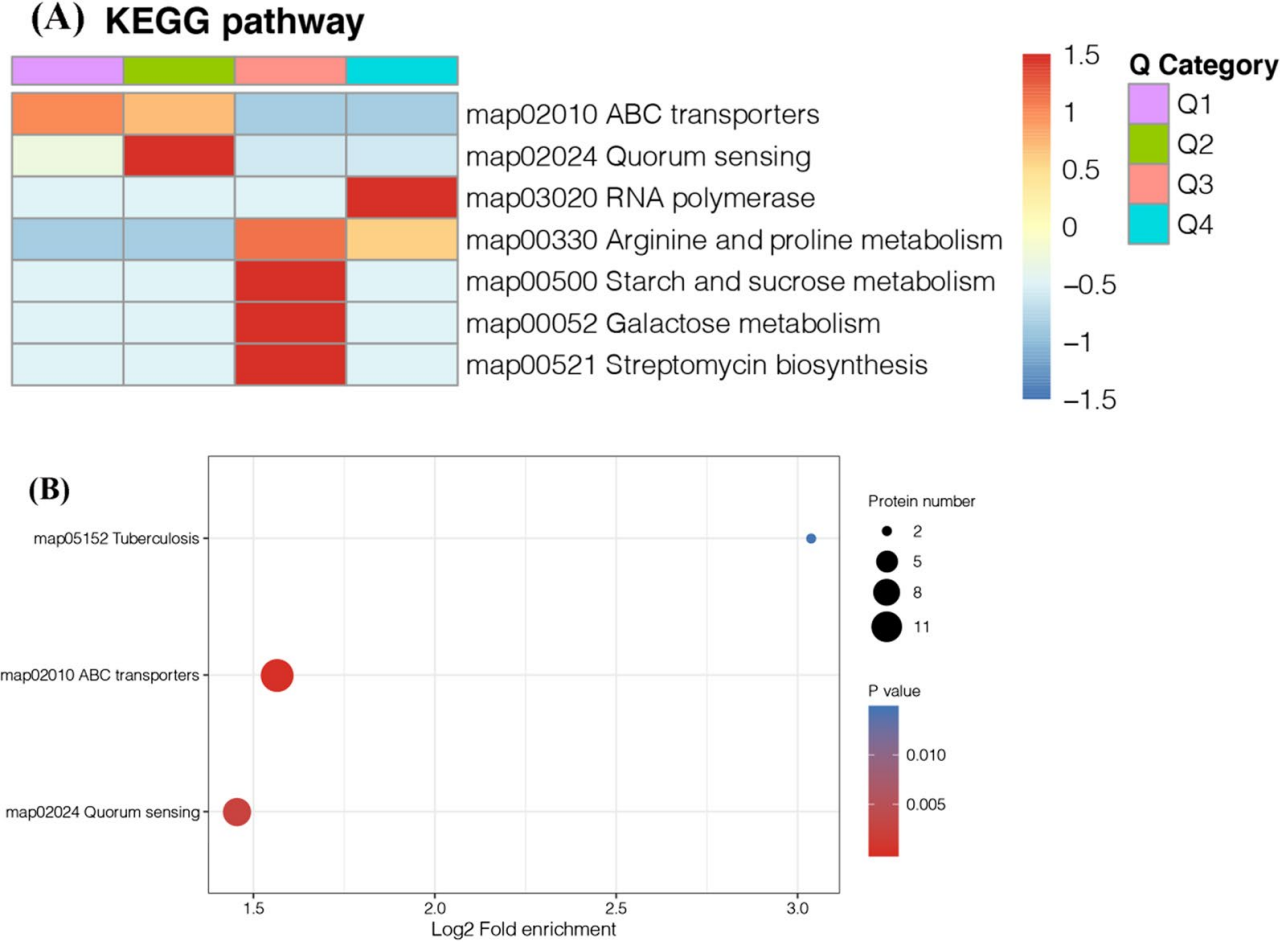
The KEGG enriched and down-regulated pathways. **A** The heatmap of KEGG pathways according to the *P* value of Fisher’s exact test obtained from enrichment analysis. The related functions were grouped together using hierarchical clustering. The horizontal axis of the heatmap represents the enrichment test results of different groups, while the vertical axis represents the differentially expressed enrichment related functions. Q1–Q4 represent differentially expressed multiples [Q1 (< 0.5); Q2 (0.5–0.667); Q3 (1.5–2); Q4 (> 2)]. **B** The bubble chart of down-regulated KEGG pathways. The circle color indicates the enrichment significance *P*-value, while the circle size is the number of different proteins in the functional class

KEGG (Kyoto Encyclopedia of Gene and Genome) is an information network that connects known molecular interactions such as metabolic pathways, complexes, and biochemical reactions. According to the proteomics data, the map02010 (ABC transporters), map02024 (Quorum Sensing), map03020 (RNA polymerase), map00330 (Arginine and proline metabolism), map00500 (Starch and sucrose metabolism), map00052 (Galactose metabolism), and map00521 (Streptomycin biosynthesis) pathways were significantly enriched (Fig. 8A). By contrast, only the map02024, map02010, and map05512 pathways were down-regulated (Fig. 8B).

The *Δdps* R12 mutant grew slower than the WT R12 strain (Fig. 4A). ABC transporter are important for the import of nutrients [39]. As shown in Additional file 4: Fig. S4A, mineral and organic ion transporters (TbpA), oligosaccharide, polyol, and lipid transporters (ChiE, BmpA and NupB), as well as phosphate and amino acid transporters (PstS, PstA, PstC, PstB, livK and TcyA) were down-regulated. ATP-dependent proteins are valuable for the removal of oxidative damage and dysfunctional proteins [43]. These results indicated that Dps may also be important for the growth of *D. wulumuqiensis*.

Bacteria cells can communicate using a cell-density dependent regulatory system known as quorum sensing (QS) [44]. QS is associated with a number of cellular processes, such as motility, biofilm formation or antibiotic production [45–47]. However, the molecular mechanisms underlying QS circuits in many bacterial species remain unclear, while QS might play important roles in the response to environmental stresses in *D. radiodurans* [43]. According to the proteomic data (Additional file 4: Fig. S3B), the QS KEGG pathway (map02024) was significantly down-regulated, and 9 proteins were mapped, including branched-chain amino acid ABC transporter substrate-binding protein (DVJ83_00750, DVJ83_02800, DVJ83_06100, and DVJ83_07370), a multifunctional fusion protein (*sec*D), and ABC transporter substrate-binding protein (DVJ83_03180, DVJ83_03190, DVJ83_04825, and DVJ83_13605). The downregulation of these proteins can also help explain the reduced stress resistance of *D. wulumuqiensis* R12 following the knockout of the *dps* gene.

### Protein–protein interaction network of differentially expressed proteins

**Fig. 9:**
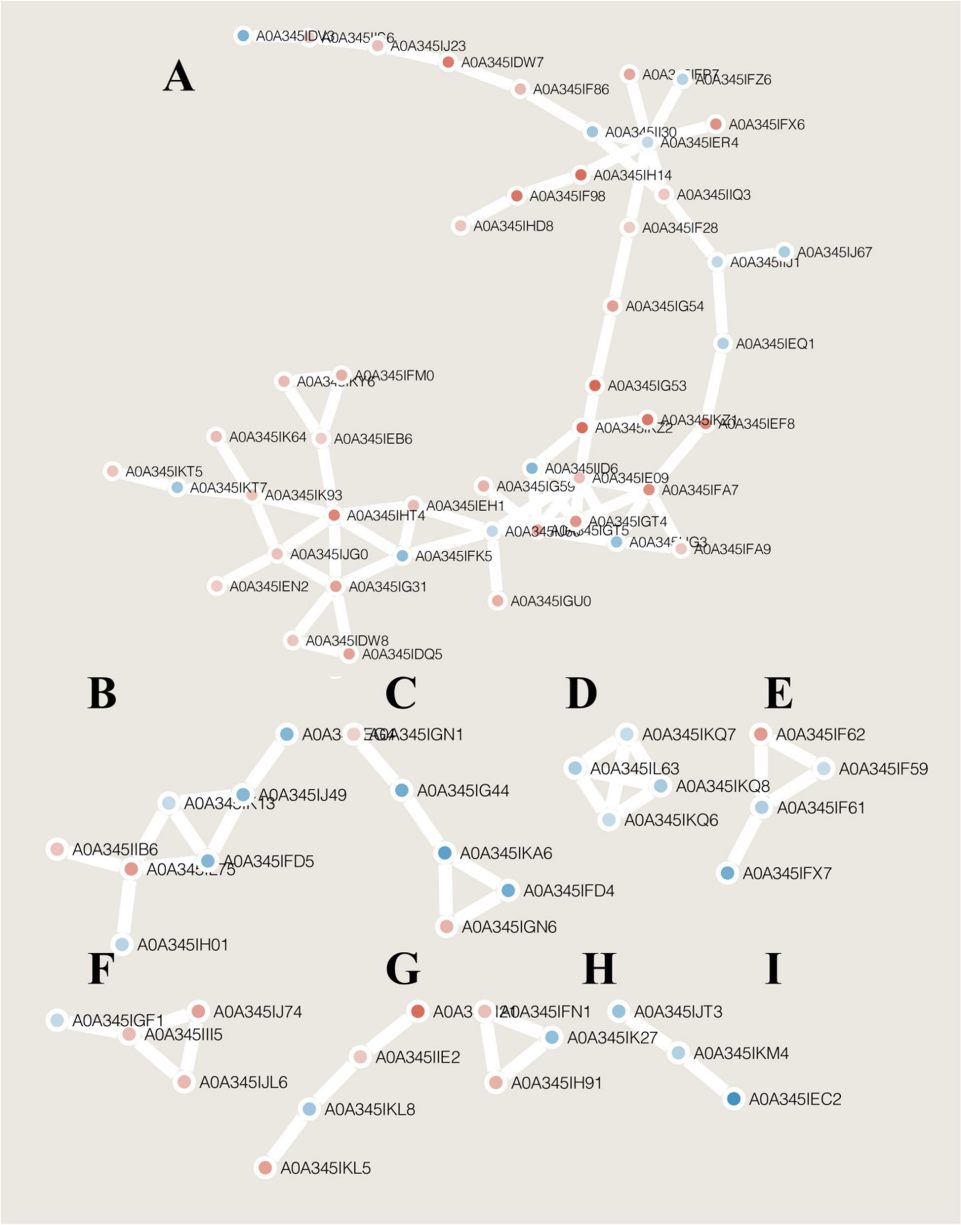
The protein–protein interaction (PPI) network of *D. wulumuqiensis* R12 revealed by functional protein association network (STRING) analysis. A total of 82 differentially expressed proteins are shown in the PPI network. The up-regulated proteins were shown in red, and down-regulated proteins in blue

Deletion of the *dps* gene in *D. wulumuqiensis* R12 induced a series of unfavorable phenotypes resulting from the interaction of multiple proteins. Cell growth and antioxidant defenses were decreased when the *dps* gene was deleted. A total of 48 up- and 34 down-regulated proteins were predicted to interact with each other. The protein–protein interaction (PPI) network was divided into 8 modules (A–I) as shown in Fig. 9. The proteins in module A are mainly involved in metabolism. Notably, glyceraldehyde-3-phosphate dehydrogenase (A0A345IFK5), which is a key enzyme in the glycolytic pathway [48], was strongly down-regulated. Dihydrolipoyl dehydrogenase (A0A345II30) is a component of the pyruvate dehydrogenase complex that plays an important role in the decarboxylation of pyruvate to produce acetyl-CoA [49]. Module D contained proteins that are part of the phosphate transport system, and down-regulated proteins were predicted to interact in pairs. Module E comprised ABC transporters, which were significantly down-regulated. Module I mainly contained antioxidative enzymes, among which superoxide dismutase (A0A345IKM4) and catalase (A0A345IEC2) were down-regulated in the PPI. These data indicated that the metabolism, transport and oxidation–reduction processes might be affected in *D. wulumuqiensis* R12 following the deletion of the *dps* gene.

## Conclusions

In this study, the sequence, structure, and function of Dps protein from *D. wulumuqiensis* R12 were analyzed. The N-terminal domain of Dps protein might play an important role in DNA binding or protection. The docking data and in vitro DNA binding experiments showed that R12 Dps has a better DNA binding ability than R1 Dps1. In addition, *Δdps* R12 mutant was constructed, and comparative proteomics was used to investigate the functions of Dps in *D. wulumuqiensis* R12. The qRT-PCR and proteomics data suggested that when the *dps* gene was knocked out in *D. wulumuqiensis* R12, the catalase gene was down-regulated. The proteomics data also suggested that that the metabolism, transport and oxidation–reduction processes were down-regulated in *D. wulumuqiensis* R12 after the *dps* gene was knocked out.

## Supplementary Information

**Additional file 1.** GO_enrichment.

**Additional file 2.** GO_terms_Level2_Classify.

**Additional file 3.** MS_identified_information.

**Additional file 4: Figure S1.** The sequencing result of WT R12 strain and *Δdps *R12 mutant. **Figure S2. **The GO analyses between *Δdps* R12 mutant and WT R12 strain. (A) The down GO term in *Δdps* R12 mutant. (B) The up GO term in *Δdps* R12 mutant. **Figure S3**. qRT-PCR of *catalase* genes in wild-type R12 strain and *Δdps *R12 mutant. The 16S rRNA was used as an internal reference gene. Error bars indicate Mean ± SD. *P value ≤ 0.05; **P value ≤ 0.01. **Figure S4. **The down-regulated KEGG pathways in the *Δdps* R12 mutation. (A) The ABC transporter KEGG pathway, and the green was down-regulated; (B) The quorum sensing KEGG pathway, and the green was down-regulated. **Table S1.** The primers used in the article. **Table S2.** List of each protein in PPI.
