# Supplementary material for: Sequence, structure, and function of the Dps DNA-binding protein from Deinococcus wulumuqiensis R12
Source: Microb Cell Fact. 2022 Jul 2;21:132. doi: 10.1186/s12934-022-01857-7 (PMC9250271; doi:10.1186/s12934-022-01857-7)
Supplement: Supplementary file 4 — Additional file 4: Figure S1. The sequencing result of WT R12 strain and Δdps R12 mutant. Figure S2. The GO analyses between Δdps R12 mutant and WT R12 strain. (A) The down GO term in Δdps R12 mutant. (B) The up GO term in Δdps R12 mutant. Figure S3. qRT-PCR of catalase genes in wild-type R12 strain and Δdps R12 mutant. The 16S rRNA was used as an internal reference gene. Error bars indicate Mean ± SD. *P value ≤ 0.05; **P value ≤ 0.01. Figure S4. The down-regulated KEGG pathways in the Δdps R12 mutation. (A) The ABC transporter KEGG pathway, and the green was down-regulated; (B) The quorum sensing KEGG pathway, and the green was down-regulated. Table S1. The primers used in the article. Table S2. List of each protein in PPI. [file 12934_2022_1857_MOESM4_ESM.docx]

**Sequence, Structure, and Function of the Dps DNA-binding protein from *Deinococcus wulumuqiensis* R12**

Yao Chen^a,b^, Zhihan Yang^b^, Xue Zhou^a^, Mengmeng Jin^a^, Zijie Dai^a^, Dengming Ming^b^, Zhidong Zhang^b,d,*^, Liying Zhu^c,*^, Ling Jiang^a,*^

^a^College of Food Science and Light Industry, State Key Laboratory of Materials-Oriented Chemical Engineering, Nanjing Tech University, Nanjing 211816, China;

^b^College of Biotechnology and Pharmaceutical Engineering, Nanjing Tech University, Nanjing 211816, China;

^c^School of Chemistry and Molecular Engineering, Nanjing Tech University, Nanjing 211816, China;

^d^Institute of Applied Microbiology, Xinjiang Academy of Agricultural Sciences/Xinjiang Key Laboratory of Special Environmental Microbiology, Urumqi, Xinjiang 830091, China;

^e^Department of General Surgery, Nanjing Drum Tower Hospital, the Affiliated Hospital of Nanjing University Medical School, Nanjing 211816, China;

^*^Corresponding authors.

Zhidong Zhang, Email: zhangzheedong@sohu.com,

College of Biotechnology and Pharmaceutical Engineering, Nanjing Tech University, Nanjing 211816, China;

Liying Zhu, Email: zlyhappy@njtech.edu.cn,

School of Chemistry and Molecular Engineering, Nanjing Tech University, Nanjing 211816, China;

Ling Jiang, Email: jiangling@njtech.edu.cn,

College of Food Science and Light Industry, Nanjing Tech University, Nanjing 211816, China.


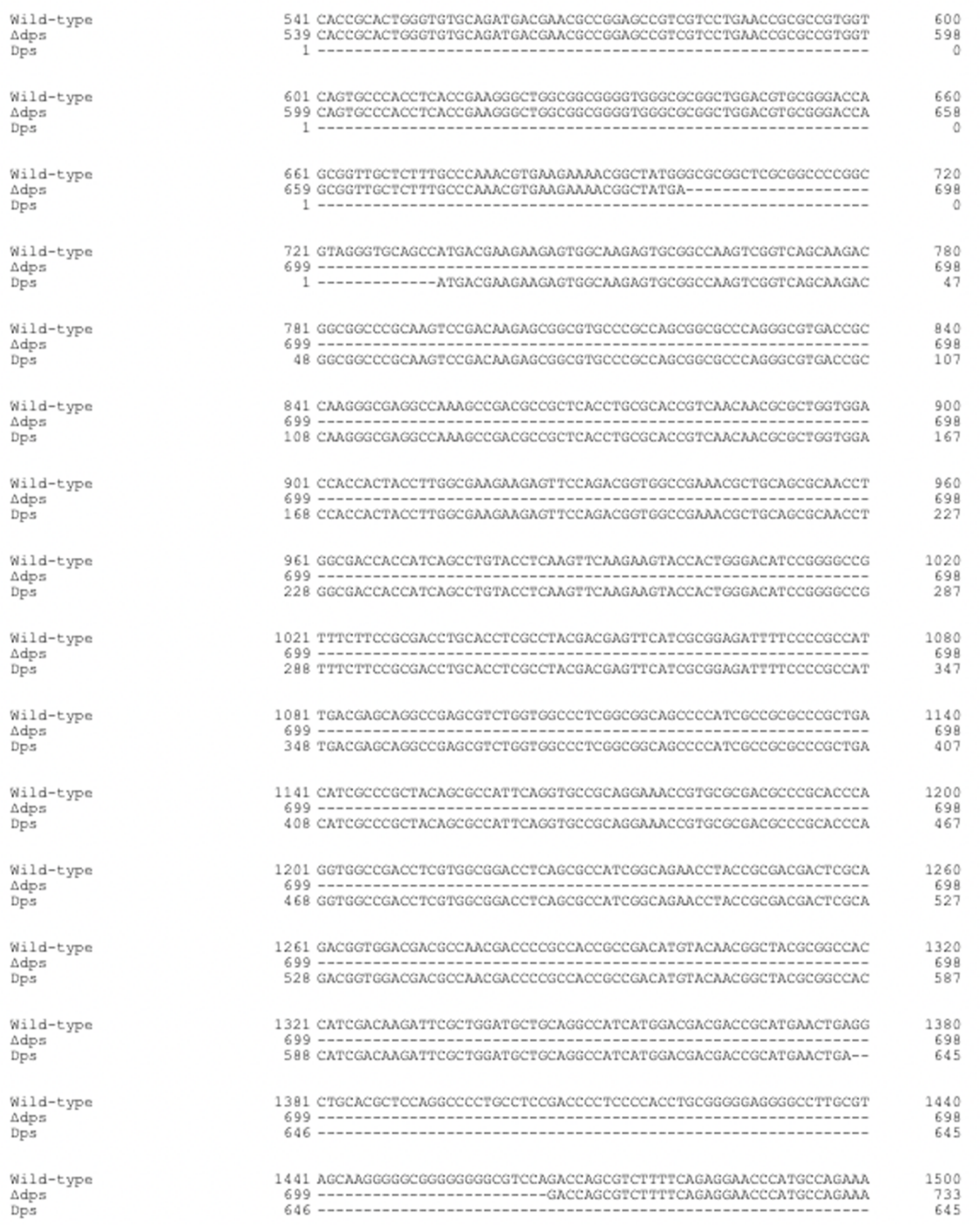


**Fig. S1.** The sequencing result of WT R12 strain and *Δdps* R12 mutant.


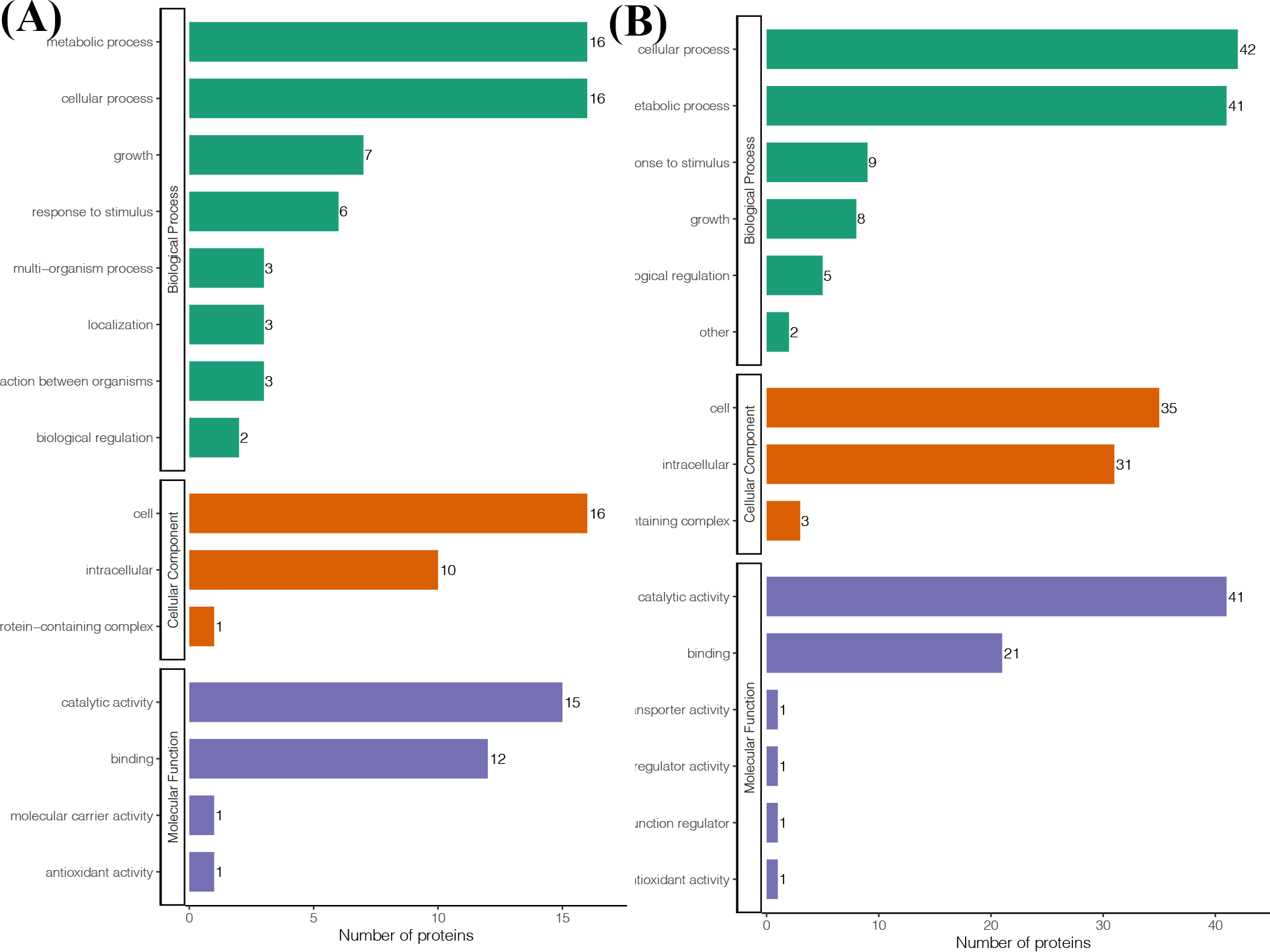


**Fig. S2.** The GO analyses between *Δdps* R12 mutant and WT R12 strain. (A) The down GO term in *Δdps* R12 mutant. (B) The up GO term in *Δdps* R12 mutant.


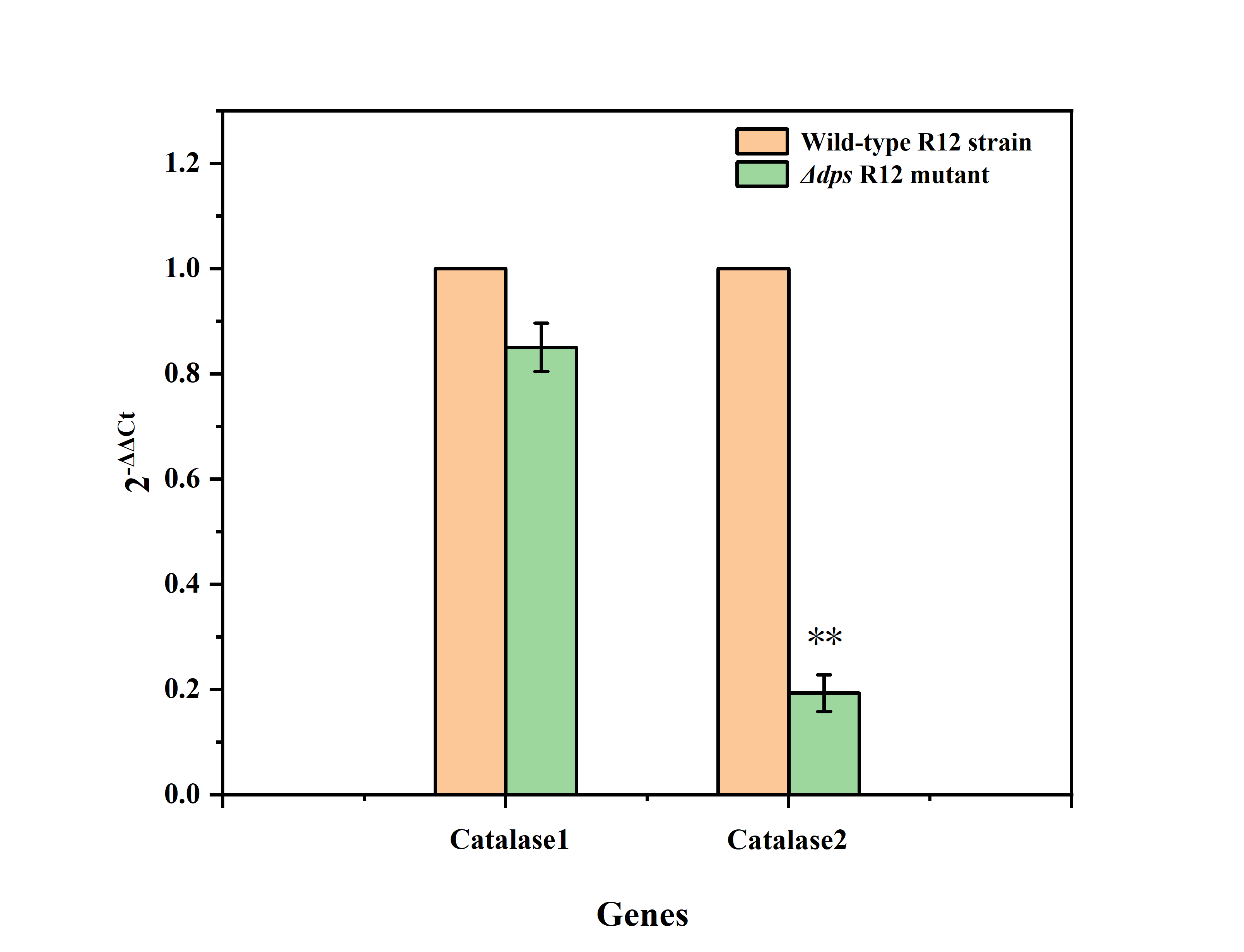


**Fig. S3**. qRT-PCR of *catalase* genes in wild-type R12 strain and *Δdps* R12 mutant. The 16S rRNA was used as an internal reference gene. Error bars indicate Mean ± SD. * *p* value ≤ 0.05; ** *p* value ≤ 0.01.


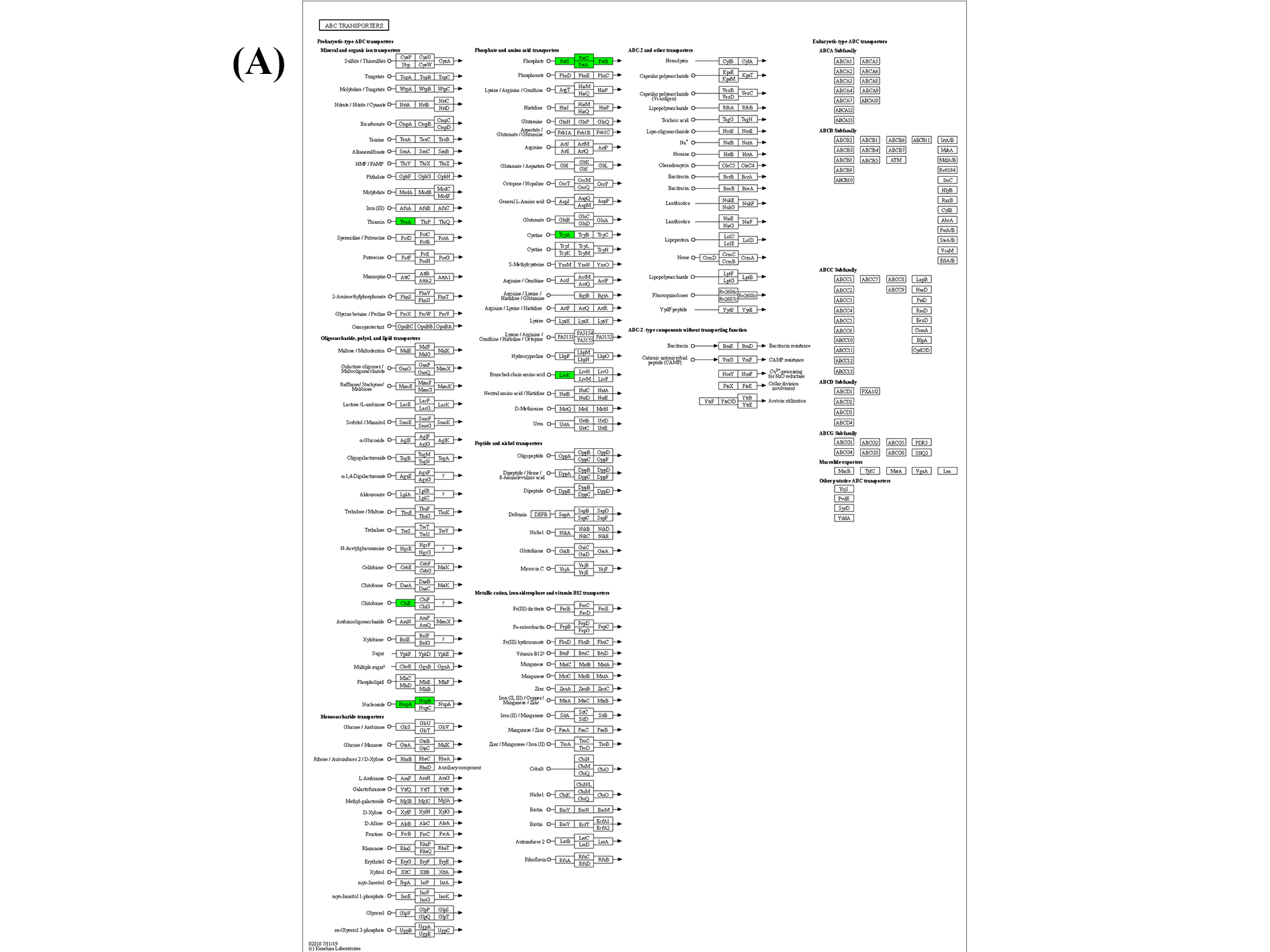

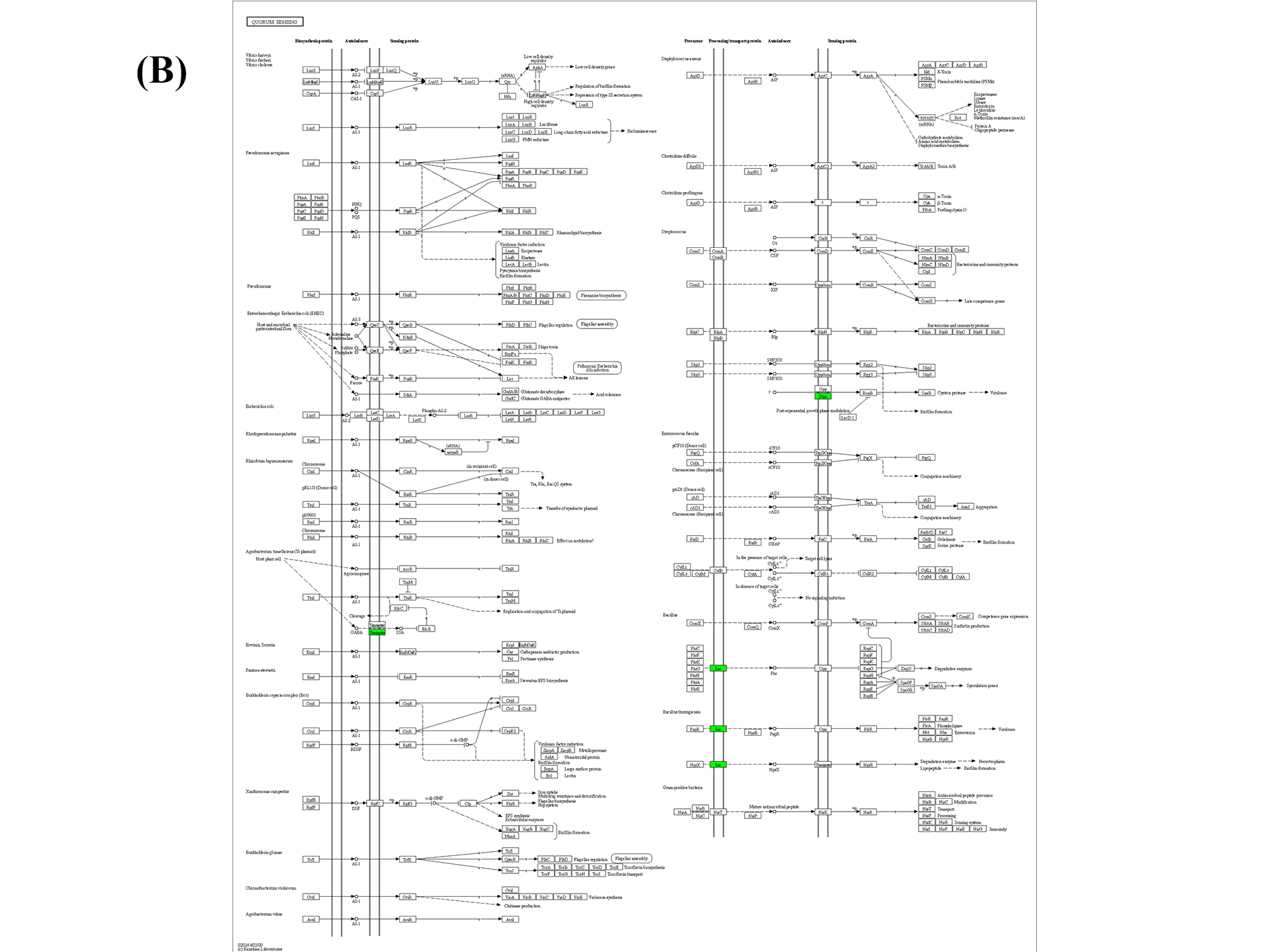


**Fig. S4.** The down-regulated KEGG pathways in the *Δdps* R12 mutation. (A) The ABC transporter KEGG pathway, and the green was down-regulated; (B) The quorum sensing KEGG pathway, and the green was down-regulated.

**Table S1. the primers used in the article**

| **Primer** | **Sequence (5’-3’)** |
| --- | --- |
| **F1** | CGGAATTCATTCTGAACACCCACAGCCAC |
| **R1** | CTCTGAAAAGACGCTGGTCTCATAGCCGTTTTCTTCACGT |
| **F2** | ACGTGAAGAAAACGGCTATGAGACCAGCGTCTTTTCAGAG |
| **R2** | CGGGATCCGTCCCATTTGGCCTTGAAGAT |
| **R12_Dps_F** | GGAATTCCATATGACGAAGAAGAGTGGCAAGAGT |
| **R12_Dps_R** | CCGCTCGAGGTTCATGCGGTCGTCGTC |
| **R1_Dps_F** | GGAATTCCATATGACGAAGAAAAGCACCAAGAG |
| **R1_Dps_R** | CCGCTCGAGGTCGAGGCGCTCGTC |
| **catalase1_F** | GCATTCAGGCCAAGGAAGC |
| **catalase1_R** | TCAGTACAGGCTGCTCGC |
| **catalase2_F** | ATGACCTACGTCCCCTACTCCC |
| **catalase2_R** | TGGCGGTCAAACGACTTGAC |
| **16S_F** | TGGCGGCGTGCTTAAGAC |
| **16S_R** | GCAAATCACCACATCACGTATTAGCT |

*** The underline represents the restriction site.**

**Table S2. List of each protein in PPI**

| **Protein accession** | **Protein description** |
| --- | --- |
| A0A345IKT5 | CoA transferase subunit A |
| A0A345IKT7 | N-acetylglucosamine-6-phosphate deacetylase |
| A0A345IK64 | Aldose 1-epimerase |
| A0A345IK93 | ROK family protein |
| A0A345IEN2 | 1, 4-alpha-glucan branching enzyme GlgB |
| A0A345IJG0 | Alpha-D-glucose phosphate-specific phosphoglucomutase |
| A0A345IHT4 | Transketolase |
| A0A345IG31 | Deoxyribose-phosphate aldolase |
| A0A345IEB6 | Aspartate-semialdehyde dehydrogenase |
| A0A345IDQ5 | Thymidine phosphorylase |
| A0A345IFM0 | Aspartokinase |
| A0A345IDW8 | Cytidine deaminase |
| A0A345IKY6 | Threonine synthase |
| A0A345IEH1 | Glycerol kinase |
| A0A345IFK5 | Glyceraldehyde-3-phosphate dehydrogenase |
| A0A345IGU0 | DNA topoisomerase (ATP-hydrolyzing) |
| A0A345IJ60 | Chaperone protein DnaK |
| A0A345IFA9 | Ribosome-recycling factor |
| A0A345IGT4 | DNA-directed RNA polymerase subunit beta |
| A0A345IFA7 | Elongation factor Ts |
| A0A345IJG3 | Elongation factor G |
| A0A345IE09 | DNA-directed RNA polymerase subunit alpha |
| A0A345IID6 | Nucleoside diphosphate kinase |
| A0A345IGT5 | DNA-directed RNA polymerase subunit beta' |
| A0A345IKZ2 | Ribonucleotide-diphosphate reductase subunit beta |
| A0A345IKZ1 | Thioredoxin |
| A0A345IG59 | Transcription elongation factor GreA |
| A0A345IEF8 | Trigger factor |
| A0A345IG53 | Sensor histidine kinase |
| A0A345IEQ1 | Multifunctional fusion protein |
| A0A345IG54 | Shikimate dehydrogenase (NADP(+)) |
| A0A345IIJ1 | Queuine tRNA-ribosyltransferase |
| A0A345IJ67 | NADPH-dependent 7-cyano-7-deazaguanine reductase |
| A0A345IF28 | 3-phosphoshikimate 1-carboxyvinyltransferase |
| A0A345IIQ3 | Thymidylate synthase |
| A0A345IKT5 | CoA transferase subunit A |
| A0A345II30 | Dihydrolipoyl dehydrogenase |
| A0A345IER4 | Glycine dehydrogenase (decarboxylating) |
| A0A345IFP7 | NADPH-dependent oxidoreductase |
| A0A345IFX6 | D-3-phosphoglycerate dehydrogenase |
| A0A345IFZ6 | Saccharopine dehydrogenase family protein |
| A0A345IH14 | Proline dehydrogenase |
| A0A345IF98 | Pyrroline-5-carboxylate reductase |
| A0A345IHD8 | Alpha/beta hydrolase |
| A0A345IF86 | Isocitrate dehydrogenase [NADP] |
| A0A345IDW7 | Adenylosuccinate lyase |
| A0A345IJ23 | Phosphoribosylformylglycinamidine synthase subunit PurL |
| A0A345IIS6 | N5-carboxyaminoimidazole ribonucleotide synthase |
| A0A345IDV3 | Lysine biosynthesis protein LysX |
| A0A345IIB6 | Acetyl-coenzyme A synthetase |
| A0A345IL75 | Malate synthase |
| A0A345IH01 | Isocitrate lyase |
| A0A345IFD5 | 2-isopropylmalate synthase |
| A0A345IK13 | Homocitrate synthase |
| A0A345IJ49 | Biotin carboxyl carrier protein of acetyl-CoA carboxylase |
| A0A345IEG4 | Acyl carrier protein |
| A0A345IGN1 | NUDIX hydrolase |
| A0A345IG44 | PEGA domain-containing protein |
| A0A345IKA6 | DUF11 domain-containing protein(predicted) |
| A0A345IGN6 | DUF4139 domain-containing protein |
| A0A345IFD4 | Permeases of the major facilitator superfamily(predicted) |
| A0A345IL63 | Phosphate transport system permease protein PstA |
| A0A345IKQ7 | Phosphate transport system permease protein |
| A0A345IKQ8 | Phosphate-binding protein |
| A0A345IKQ6 | Phosphate import ATP-binding protein PstB |
| A0A345IF62 | ABC transporter ATP-binding protein |
| A0A345IF59 | ABC transporter substrate-binding protein |
| A0A345IF61 | ABC transporter permease |
| A0A345IFX7 | ABC transporter substrate-binding protein |
| A0A345IGF1 | Cysteine-tRNA ligase |
| A0A345III5 | Arginine-tRNA ligase |
| A0A345IJ74 | Valine-tRNA ligase |
| A0A345IJL6 | Lysine-tRNA ligase |
| A0A345IKL5 | Histidine phosphatase family protein |
| A0A345IKL8 | Acyl-CoA dehydrogenase |
| A0A345IIE2 | Fatty acid-CoA ligase |
| A0A345II21 | Acyl-CoA dehydrogenase |
| A0A345IFN1 | DUF3459 domain-containing protein |
| A0A345IH91 | Glycoside hydrolase family 13 protein |
| A0A345IK27 | Alpha-amylase |
| A0A345IJT3 | Type 1 glutamine amidotransferase domain-containing protein |
| A0A345IKM4 | Superoxide dismutase |
| A0A345IEC2 | Catalase |
